# The Effect of Grapevine Age (*Vitis vinifera* L. cv. Zinfandel) on Phenology and Gas Exchange Parameters over Consecutive Growing Seasons

**DOI:** 10.3390/plants10020311

**Published:** 2021-02-05

**Authors:** Vegas Riffle, Nathaniel Palmer, L. Federico Casassa, Jean Catherine Dodson Peterson

**Affiliations:** Wine and Viticulture Department, California Polytechnic State University, San Luis Obispo, CA 93407, USA; vriffle@calpoly.edu (V.R.); nrpalmer@calpoly.edu (N.P.); lcasassa@calpoly.edu (L.F.C.)

**Keywords:** old vine, Zinfandel, phenology, gas exchange, Central Coast of California

## Abstract

Unlike most crop industries, there is a strongly held belief within the wine industry that increased vine age correlates with quality. Considering this perception could be explained by vine physiological differences, the purpose of this study was to evaluate the effect of vine age on phenology and gas exchange parameters. An interplanted, dry farmed, Zinfandel vineyard block under consistent management practices in the Central Coast of California was evaluated over two consecutive growing seasons. Treatments included Young vines (5 to 12 years old), Control (representative proportion of young to old vines in the block), and Old vines (40 to 60 years old). Phenology, leaf water potential, and gas exchange parameters were tracked. Results indicated a difference in phenological progression after berry set between Young and Old vines. Young vines progressed more slowly during berry formation and more rapidly during berry ripening, resulting in Young vines being harvested before Old vines due to variation in the timing of sugar accumulation. No differences in leaf water potential were found. Young vines had higher mid-day stomatal conductance and tended to have higher mid-day photosynthetic rates. The results of this study suggest vine age is a factor in phenological timing and growing season length.

## 1. Introduction

Grapevines (*Vitis vinifera* L.) are long-lived perennial plants, with one such vine documented as more than 400 years old [1,2]. However, under commercial conditions, vineyards are typically productive for 30 to 50 years. Although the specific number of years required to make a vineyard block economically viable varies from site-to-site and by marketing goal, the longer vines are kept in production, the larger the profit margin. Many factors have contributed to decreasing lifespan of commercial vineyards, including damage and decline phylloxera (*Daktulospaira vitifoliae* Fitch) [3], various nematode species [4], and wood rot diseases, such as *Eutypa lata* [5]. Although many European vineyards were replated due to the introduction of phylloxera in 1863, Australia and California still maintain vineyards with planting dates going back to the mid-1800s [2,6]. Old vine vineyards are highly regarded in both regions, with organizations developed specifically to preserve old vine heritage [7,8]. Although the time at which vines are designated as “old” is somewhat unclear, most agree a decreased capacity to set and mature fruit is a common factor [9,10]. This, in turn, is thought to result in more concentrated flavors, yielding superior fruit and wine quality [10].

It is important to note wine quality perception could be the result of vineyard management technique, rather than vine age. For example, young vineyards are generally grafted onto rootstocks with available irrigation, while old vineyards are generally ungrafted with little to no irrigation under dry farmed conditions [11]. Nonetheless, as a result of the rarity, production difficulty, and perceived enhancement of wine quality, “old” vines have become increasingly sought after and valued by industry and consumers [12]. Not only does an “old vine” wine label yield higher prices in the market, but empirical accounts also suggest older vineyards typically demand a high price per ton. This trend is despite the fact there is currently no legally recognized definition of what constitutes an “old” vine. The term “old vine” pervades the wine labels of many cultivars; however, this study evaluates *Vitis vinifera* L. cv. Zinfandel. This European cultivar was selected for its prevalence in the “old vine” wine market and deep ties to California viticulture, specifically in the Central Coast [13]. The California Central Coast American Viticultural area (AVA) spans from northern-most San Francisco to southern-most Santa Barbara [14], an area with a wide range of environmental variables and conditions. 

Due to the influence of climate change on grape growing [15,16], regional-based research on vine growing season length, water-use efficiency, and gas exchange is vital. Grapevine phenology tracks the progression of key developmental stages through a growing season. While the timing of these stages is primarily temperature driven [17], other abiotic factors can influence vine phenology such as cultivar, soil properties, slope orientation, and precipitation [18,19,20,21]. While no studies to our knowledge have evaluated the effect of vine age in Zinfandel grapes and wines in the Central Coast of California, a handful of studies have evaluated the effect of vine age on vine performance in other regions and cultivars [2,9,22,23,24,25,26,27]. No differences in phenological shifts between grapevine age groups have been found [28]. Results for the effect of vine age on sugar content at harvest are contradictory, with some studies reporting little to no differences between young and old vines [6,28,29], and others reporting lower sugar content at harvest for old vines [24]. Younger vines have been reported as more sensitive to water stress conditions, attributed to less developed root systems [23,27]. Research on diurnal vine gas exchange as a function of age is lacking, but young vines have been reported to show relatively lower photosynthesis [23], stomatal conductance, and transpiration compared to old vines [27]. 

A common thread in comprehensive vine age studies is the difficultly to separate seasonal variability and confounding variables from vine age effects [6,23,24,28]. In order to minimize these effects, this study was performed at a single interplanted vineyard block with young (5 to 12 years old) and old (40 to 60 years old) vines, under uniform management practices. The purpose of this study is to evaluate the influence of grapevine age on phenology and gas exchange parameters in the Central Coast of California. This study serves to lay a foundation from which the industry can understand and interpret vine growth and variation as a function of age. 

## 2. Results

### 2.1. Climate Data

**Figure 1:**
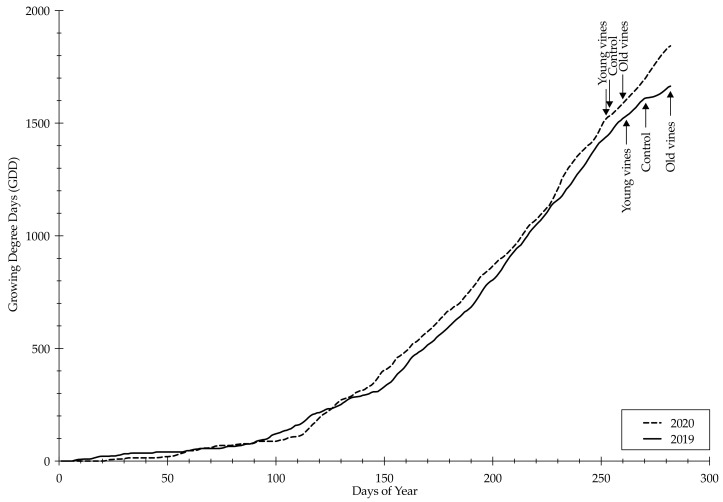
Growing degree days (GDD) accumulation for Atascadero, California weather station 163 during the 2019 and 2020 growing season. Harvest dates for each treatment marked with an arrow.

While the 2019 and 2020 growing season are classified within the same Winkler region (III), there was a 237.6 GDD difference between the two seasons (Table 1). Based on this difference, the 2020 growing season was considerably warmer. As well, the 2020 growing season had a lower annual precipitation but a higher seasonal precipitation compared to the 2019 growing season. During the growing season, there were 34 days with maximum temperatures above 35 °C in 2020 versus 30 days in 2019. Furthermore, there were six days with maximum temperatures above 40 °C in 2020 versus zero days in 2019. The six days in 2020 correlated to two excessive heat waves, both of which occurred after véraison. The average and maximum air temperature was higher in May, June, August, September, October for the 2020 growing season as compared to 2019 growing season (Table 2). Due to differences in sugar accumulation, the harvest of the Young vine treatment occurred nine days before the harvest of the Control treatment and 21 days before the harvest of the Old vine treatment during the 2019 growing season (Figure 1). Contrastingly, during the warmer 2020 growing season, the harvest of the Young vine treatment occurred two days before the harvest of the Control treatment and nine days before the harvest of the Old vine treatment (Figure 1).

### 2.2. Phenology and Senescence Tracking

**Figure 2:**
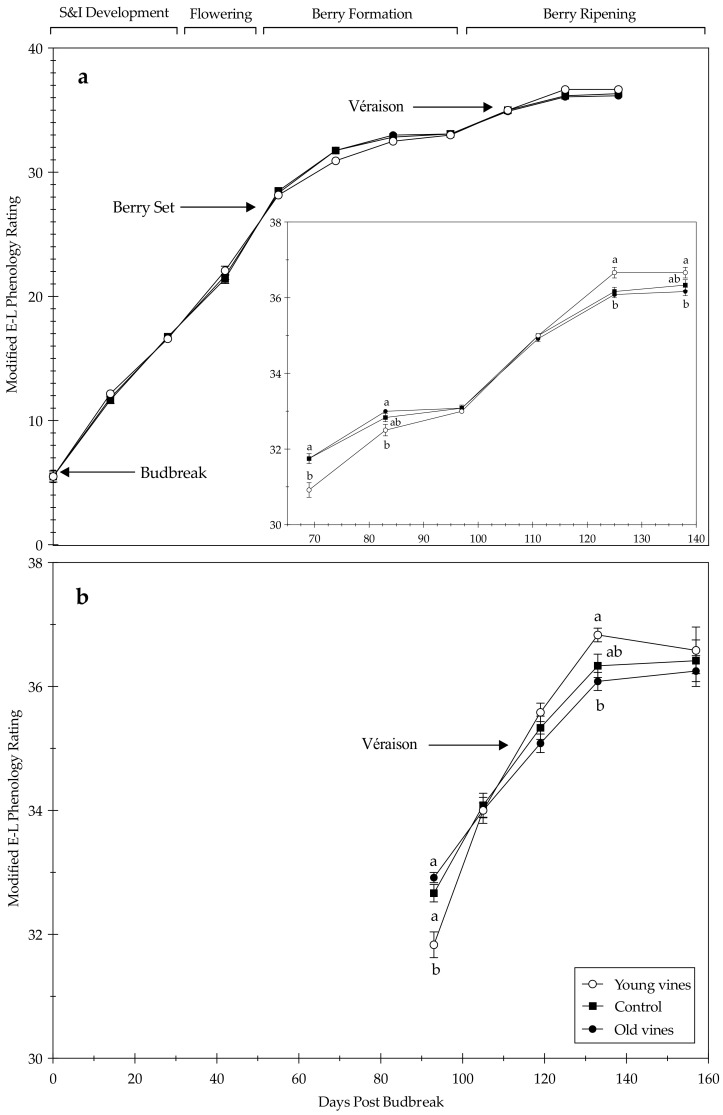
Phenology tracking during the (**a**) 2020 and (**b**) and 2019 growing season (*n* = 12). One-way analysis of variance (ANOVA) showing treatment means, with bars representing the standard error of the mean. Different letters indicate differences between treatment groups based on Tukey HSD. Graph inset enlarged the 2020 data from two weeks post-berry set to four weeks post-véraison. Key phenological stages (budbreak, berry set, and véraison) and general growth labels (shoot and inflorescence development, flowering, berry formation, and berry ripening) are marked based on the Modified Eichhorn-Lorenz (E-L) system. S&I development corresponds to shoot and inflorescence development.

All treatments were evaluated during the growing season based on the Modified Eichhorn-Lorenz (E-L) system. In the 2019 growing season, there was a difference in phenological rating between treatments at berry ripening and two weeks post-véraison (Figure 2; *p ≤* 0.0001 and *p* = 0.0050, respectively). While Young vines lagged slightly behind during berry ripening, Old vines lagged slightly behind at two weeks post-véraison. These trends, while not statistically significant, were also observed at two weeks post-berry ripening, véraison, and four weeks post-véraison. While the Control treatment was not statistically different from the Old vine treatment at berry ripening, it was numerically lower (rating 32.7 compared to rating 32.9). This trend was observed at every growth stage in the 2019 growing season.

In the 2020 growing season, there were no statistical differences in phenological rating from budbreak to berry set. There were differences at two weeks post-berry set, four weeks post-berry set, two weeks post-véraison and four weeks post-véraison (*p* = 0.0005, *p* = 0.0087, *p* = 0.0021 and *p* = 0.0365, respectively). While Young vines lagged slightly behind at two weeks post-berry set and four weeks post-berry set (otherwise called berry ripening), Old vines lagged slightly behind at two weeks post-véraison and four weeks post-véraison. Compared to Old vines, Young vines progressed slowly during berry formation but quickly during berry ripening during the 2019 and 2020 growing seasons. During the 2020 growing season, the Control treatment was statistically different from Young vine treatment but statistically similar to Old vine treatment at two weeks post-berry set and two weeks post-véraison. The Control treatment was statistically similar to both treatments at four weeks post-berry set and four weeks post-véraison. While the treatments were not significantly different at any of the other growth stages (budbreak, bloom, berry set, berry ripening, and véraison) in the 2020 growing season, it is important to note the inconsistency in numerical ratings where the Control treatment was not measured as the intermediate between Young vine and Old vine treatments. 

In the 2020 growing season, all treatments were evaluated post-harvest based on the Dodson Walker Senescence Scale [30]. Young vines had a higher leaf chlorosis rating than Old vines during the onset of senescence, with Young vines showing less than or equal to 25% leaf chlorosis symptoms (*p* = 0.0197) (Table 3). This trend continued into the next data collection date (10/30/20), although Young vines rated statistically similar to Old vines in leaf chlorosis ratings (*p* = 0.0437) (Table 3). Leaf chlorosis progressed quickly into the next data collection date (11/13/20), with all treatments rated at 100% leaf chlorosis (Table 3). There were no statistically significant differences in leaf abscission at any of the data collection dates. However, Young vines tended to progress quicker up until the third data collection date (11/13/20), where Old vines tended to rate higher compared to Young vines (Table 3). This trend was observed a week later (11/22/20).

### 2.3. Leaf Water Potential and Gas Exchange Measurments

In the 2019 growing season, there were no differences between treatments in mid-day Ψ_leaf_ measurements at berry formation or one week post-véraison (Table 4). In the 2020 growing season, there were no differences between treatments in pre-dawn and mid-day Ψ_leaf_ measurements at véraison or four weeks post-véraison (Table 5). Two consecutive days at véraison were measured due to significant differences in maximum air temperatures. While no significant differences in pre-dawn or mid-day Ψ_leaf_ measurements were found between treatments at either of these dates, Young vines had a slightly lower readings compared to Old vines (Table 5). All treatments recovered from the first day heatwave (véraison day 1), with mid-day Ψ_leaf_ measurements lowering from high-moderate stress to moderate-low stress on véraison day 2 (Table 5). Significant differences in mid-day and pre-dawn Ψ_leaf_ measurements were found between growth stages (*p* ≤ 0.0001 and *p* = 0.0111, respectively) (Table 5).

**Figure 3:**
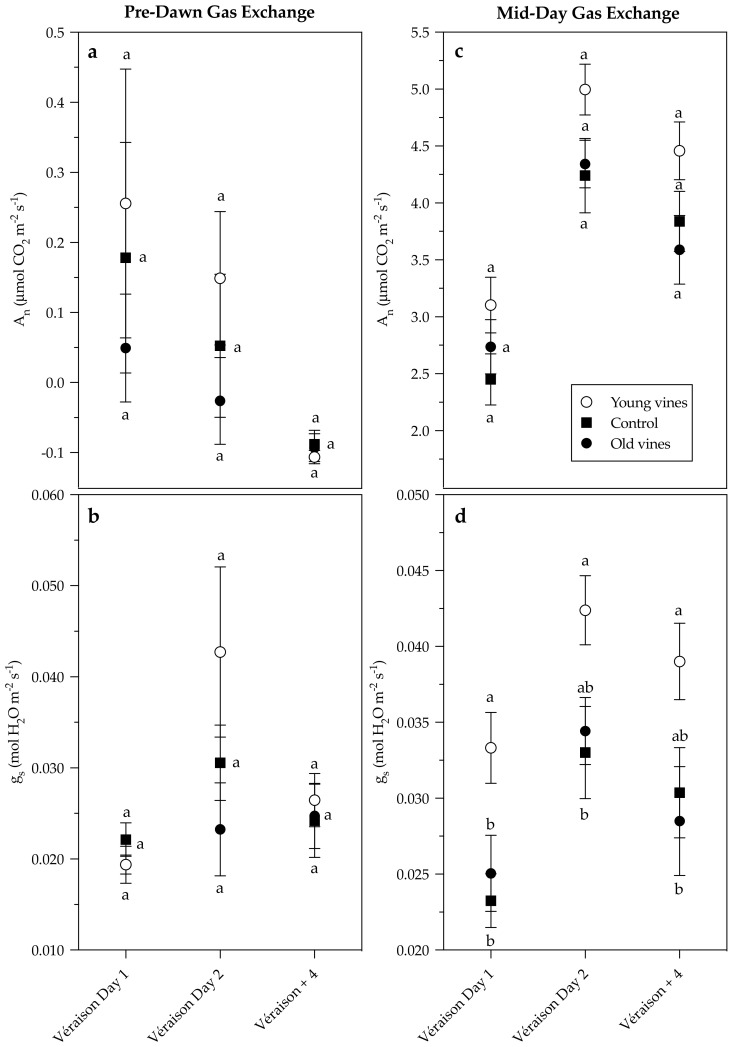
Gas exchange parameters during the 2020 growing season showing (**a**) pre-dawn photosynthetic rate (A_n_), (**b**) pre-dawn stomatal conductance (g_s_), (**c**) mid-day photosynthetic rate (A_n_), and (**d**) mid-day stomatal conductance (g_s_) (*n* = 15 for mid-day). Outliers were excluded from pre-dawn measurements (*n* = 5 for Young vines at véraison day 2 and Control vines at véraison + 4; *n* = 6 at véraison day 1). Véraison + 4 corresponds to four weeks post-véraison. Two-way analysis of variance (ANOVA) showing treatment means, with bars representing the standard error of the mean. Different letters indicate significant differences between treatment groups based on Tukey HSD. Different scales are displayed on the *Y*-axis of each figure. Treatment (T), growth stage (G), and T × G interactions were analyzed (*p*-values reported in Section 2.3).

**Figure 4:**
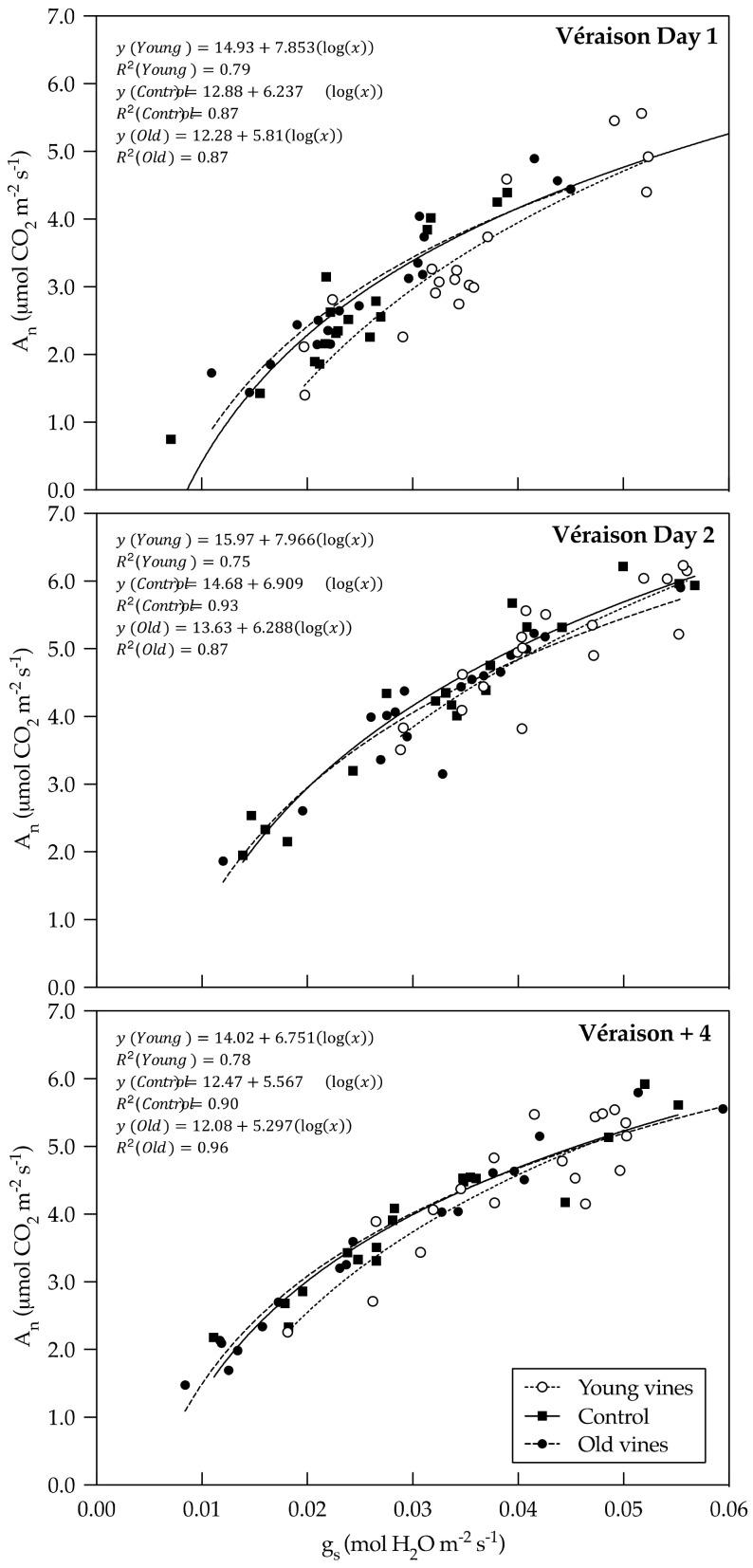
Relationships between mid-day photosynthetic rate (A_n_) and stomatal conductance (g_s_) at different growth points in the 2020 growing season. Véraison + 4 corresponds to four weeks post-véraison. Curves of logarithmic regressions of Young and Old vines are significantly different on Véraison day 1 (*p* = 0.0152).

There were no statistical differences in pre-dawn or mid-day photosynthetic rates (A_n_) between treatments at véraison or four weeks post-véraison; however, young vines tended to have notably higher photosynthetic rates (Figure 3). Young vines had higher mid-day stomatal conductance (g_s_) measurements than Old vines at véraison day 1 and four weeks post-véraison (*p* = 0.0058 and *p* = 0.0440, respectively) (Figure 3). While not statistically different, Young vines tended to have higher pre-dawn stomatal conductance measurements as well (Figure 3). Importantly, outliers were excluded from pre-dawn measurements (*n* = 5 for Young vines at véraison day 2 and Control vines at véraison +4; *n* = 6 at véraison day 1) which led to large standard error of the mean values (Figure 3). Significant differences in pre-dawn photosynthetic rates and stomatal conductance measurements were found between growth stages (*p* = 0.0159 and *p* = 0.0042, respectively). Mid-day photosynthetic rates were affected by treatment and growth stage, although no treatment × growth stage interaction was found (*p* = 0.0021 and *p* ≤ 0.0001, respectively). Mid-day stomatal conductance measurements were affected by treatment and growth stage, although no treatment × growth stage interaction was found (*p* ≤ 0.0001 and *p* = 0.0001, respectively). Logarithmic regression analysis of the relationship between stomatal conductance and Ψ_leaf_ measurements at both pre-dawn and mid-day found no correlation. Logarithmic regression analysis of the relationship between mid-day photosynthetic rates and stomatal conductance measurements found Young vines are statistically different from Old vines at véraison day 1 (*p* = 0.0115), which was the warmest of the two véraison dates (Figure 4). There were no regression differences in mid-day photosynthetic rates and stomatal conductance measurements between treatments at véraison day 2 or four weeks post-véraison (Figure 4).

## 3. Discussion

This study was conducted during the 2019 and 2020 growing season with the aim to determine the effects of vine age on physiological timing and processes of cv. Zinfandel vines grown in the Central Coast of California (USA), which has been historically planted in California since 1850 [10,13]. Young vines (5 to 12 years old) and old vines (40 to 60 years old) were compared, with a Control treatment (a mix of both Young and Old vines) representing the vineyard block.

It is widely accepted that temperature affects phenology, berry quality, and berry ripening [31,32]. While the difference in growing degree days (GDD) accumulation during the 2019 and 2020 season was not great enough to change the Winkler Index region classification (Region III), there was a 237.6 GDD difference in favor of the 2020 growing season (Table 1). A 214 GDD increase correlates to a 1 °C increase in mean growing season temperature; therefore, the 2020 growing season was approximately 1.1 °C warmer than the 2019 growing season. Generally, the optimum temperature range for vegetative growth in grapevines is 25 °C to 35 °C [33]. Not only was the 2020 growing season characterized by greater GDD accumulation, but there were also two excessive heat waves (above 40 °C) after véraison (Figure 1). Considering temperature is the most important climatic factor influencing viticulture [17], these events most likely influenced phenological timing of the vines on the experimental site. Indeed, warmer temperatures are needed for vine physiological activity and subsequent sugar accumulation [32]; however, critical heat wave temperatures can cause vine physiological shutdown which stops sugar accumulation [34,35].

**Figure 5:**
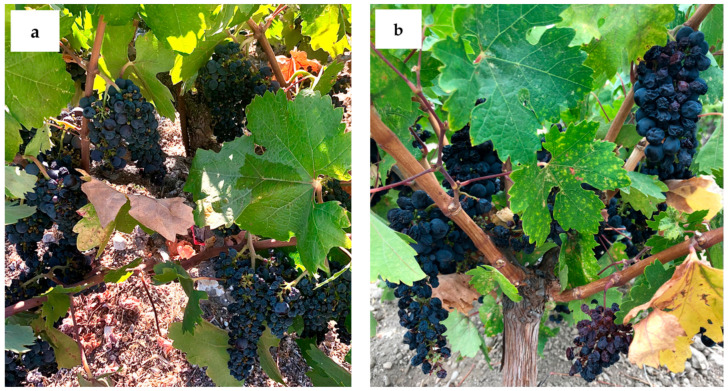
Berry water loss and sunburn symptoms in Young vines during the (**a**) 2019 and (**b**) 2020 growing season. Symptoms were pictured on 20 September 2019 (43 days post-véraison) compared to 2 September 2020 (34 days post-véraison). The second heatwave in 2020 occurred two days after this picture was taken.

While critical temperatures inhibit photosynthesis, increased sugar concentration from these events is attributed to evaporative loss from berries [35,36]. Berry water loss and sunburn symptoms were observed during both seasons, but these symptoms were more prominent after the 2020 heatwave events (Figure 5). Furthermore, these symptoms were expressed earlier in the 2020 growing season (Figure 5). These symptoms could be minimized by canopy size [37]. Differences in canopy size have been reported, with young vines displaying smaller canopy size up until the fourth year after planting compared to old vines [27]. This trend needs to be further investigated on cv. Zinfandel vines. The difference in harvest dates between the 2019 and 2020 growing season was most likely due to both extreme temperatures, water stress, and GDD accumulation (Figure 1). Additionally, the difference in harvest dates between treatments could be attributed to a greater tolerance for high temperatures in older vines, possibility due more extensive root systems [2,23,27] or greater canopy size [2,27].

Previous studies have reported no differences phenological shifts between grapevine age groups [28]. The timing of budbreak and flowering has been shown as consistent within a cultivar, while the timing of véraison and maturity is less predictable due to variability in management practices [38]. This trend was observed in our study, in that vine age was not found to be a determining factor in phenological development from budbreak to berry set. Stored carbohydrate reserves in permanent woody tissues are essential for early season growth following budbreak, which suggests in the present study, the reserves in Young and Old vines may be the same. In both growing seasons, Young vines progressed slower than Old vines during berry formation (berry set + 2 weeks to berry set + 4 weeks) (Figure 2). However, Young vines progressed quicker than Old vines during berry ripening and reached maturity quicker (Figure 2). Considering abiotic factors influence vine phenology such as soil properties [18,19], slope orientation [20], and precipitation [21] have been minimized by site selection, these trends suggest that vine age in cv. Zinfandel grapevines influences the timing of phenological events primarily after berry set and onwards. Whether the differences in berry formation and ripening is either a function of vine age, a result of the effect of vine age on vine yield, or a combination thereof, is an important distinction. The ripening delay exhibited by Old vines could be a result of higher yield and, by extension, a greater source to sink ratio, when compared to Young vines (Table S1). A greater source to sink ratio, in the context of berry ripening, translates into an inadequate amount of photosynthate production from the leaves (source) to support increasing sugar accumulation in the fruit (sink) [39,40]. This could explain the difference in harvest dates, considering Old vines progressed slower than Young vines during berry ripening. While the reasons for this trend in this study are unclear, these findings are contradictory to previous studies which found little to no differences in sugar content at harvest between young and old vines [6,28,29]. Differences in harvest dates and growing season length serve as a tool for wine growers in California; for example, younger vineyards may be less likely to be affected by late-season heatwaves [40], or the increased pressure due to wildfires and potential risk of smoke taint in the finished wines. Furthermore, these differences could warrant tailored management practices in vineyards which have diverse age groups, especially those that are interplanted [28]. The implications of harvest date differences on subsequent wine quality deserve further analysis. For example, shorter intervals from budbreak to harvest have been correlated with enhanced wine quality [17], while longer intervals can increase phenolics and aromatic ripeness [41]. The results herein presented demonstrate that vine age in cv. Zinfandel grapevines could be a factor in growing season length and phenological timing in the Central Coast of California.

Comparing the timing of leaf senescence based on vine age provides further insight into growing season length. As would be assumed based on phenological progression, Young vines expressed leaf chlorosis and abscission symptoms before Old vines. However, Old vines progressed quickly in November, and rated slightly higher in leaf chlorosis symptoms. Abscission ratings were equal between Young and Old vines in November of 2020, which suggests there were no differences in progression to dormancy. While senescence differences occurred, the implications of this trend are slight considering there were no differences in budbreak timing. Further analysis is needed over multiple growing seasons to determine the validity of these differences.

The influence of vine age on vine gas exchange parameters and water status is particularly important due to climate change trends, California irrigation restrictions [42], as well as the prevalence of dry farmed old vine vineyards in California. Previous research found that younger vines are more sensitive to water stress conditions than older vines, possibly due to less developed root systems [23,27]. However, these studies were performed on different cultivars grown in different wine growing regions. No significant differences in pre-dawn Ψ_leaf_ measurements were found between treatments at any phenological date in this study. Additionally, no differences in mid-day Ψ_leaf_ measurements were found between treatments in either growing season (2019 and 2020). Two consecutive days were measured at véraison, with the first day registering high to moderate stress mid-day Ψ_leaf_ measurements as a result of warm temperatures [43]. Both Young and Old vines recovered on the cooler second day, registering moderate to low stress mid-day Ψ_leaf_ measurements. While not statistically significant, Young vines had lower pre-dawn and mid-day Ψ_leaf_ measurements compared to Old vines on these two consecutive véraison dates, suggesting higher susceptibility to temperature extremes and water stress. This trend was also seen with mid-day Ψ_leaf_ measurements in the 2019 growing season; however, said differences in both seasons were minimal with about 0.1 megapascal (MPa) difference between Young and Old vine Ψ_leaf_ measurements. 

Previous studies have found lower photosynthesis [23], stomatal conductance, and transpiration in young vines compared to old vines [27]. However, these differences were dependent on seasonal factors [27]. In the present study, Young vines showed higher mid-day stomatal conductance measurements, and tended to have higher photosynthetic rates. Furthermore, logarithmic regression analysis of the relationship between mid-day photosynthetic rates and stomatal conductance indicated Young vines were statistically different from Old vines at véraison day 1, which was the warmest of the two dates. In terms of trends, at lower rates of stomatal conductance (≤0.04 mol H_2_O m^−2^ s^−1^), Old vines tended to display higher photosynthetic rates. In other words, Old vines showed higher intrinsic water-use efficiency (A_n_/g_s_) compared to Young vines. However, Young vines tended to have higher stomatal conductance and higher maximum photosynthesis which suggests stomata remained more open compared to Old vines. Considering stomatal closure is one of the earliest responses to water deficit, greater stomatal conductance in Young vines coupled with a tendency for lower Ψ_leaf_ could suggest a different response to water stress. Old vines displayed near-isohydric stomatal response to elevated water stress. Conversely, Young vines displayed a more near-anisohydric stomatal response through continued transpiration and photosynthesis at a higher level compared to Old vines. However, other factors have been shown to influence stomatal closure, including cultivar [44] and xylem vessel size [45]. Nonetheless, in the context of the present study, the effect of these factors on vine age are unlikely because the treatments have the same genotypes. Some studies have suggested rooting depth contributes to conductance capacity and sensitivity to xylem embolism [46], resulting in stomatal closure through a hydraulic signal involving abscisic acid (ABA) [44,45,47], while others have suggested that new root production, rather than permanent root structures, better explain this phenomenon [48]. This suggests that differences in rooting pattern between Young and Old vines could play a part in stomatal closure. Further investigation, with larger sample sizes, is needed to determine the influence of seasonal factors. While the reasons are unclear, the véraison day 1 regression analysis displayed a difference in physiological behavior during heat events. Studies have found heat events decrease mid-day photosynthetic activity and increase mid-day stomatal conductance in Riesling and Malbec [35,49]. Decreased mid-day photosynthetic activity across all treatments was herein seen in véraison day 1 compared to véraison day 2. No increase in mid-day stomatal conductance between the two dates was observed. While the effects of heat waves can indeed be minimized by adequate irrigation, which enables evaporative cooling [35,49], such applications are not practically feasible in dry farmed old vine vineyards. Further investigation into the architecture and distribution of Young and Old vine root systems is needed to determine the reasons for observed water and heat stress differences.

## 4. Materials and Methods

### 4.1. Site Description and Experimental Design

This study was initiated on June 2019 at a commercial vineyard in San Luis Obispo county, California, USA (35°34′07.9″ N–120°42′14.7″ W), which is located in the Templeton Gap District AVA. The vineyard is dry farmed, head-trained spur-pruned, and conventionally managed with 2.44 × 2.44 m vine spacing. The dominant soil series is Lockwood channery loam, characterized by an alluvial fan, with a 0 to 2% slope [50]. A small portion of the vineyard, which was included in the experimental design in order to increase the sample size of Young vines, is on a 9 to 15% slope with similar soil texture and parent material [50]. Soil core tests in the experimental block indicated loam to silt loam soils. The experimental block consists of both own-rooted vines (*Vitis vinifera* L. cv. Zinfandel) and replants from the same source material. When older vines were determined to be unproductive, genetically identical scion plant material was grafted onto St. George (*Vitis rupestris* Scheele) rootstock. This rootstock difference was determined negligible for purposes of the experiment due to similarities in *Vitis vinifera* and *Vitis rupestris* root architecture and grape quality contributions [51]. A completely randomized design was established, with Young vines classified as 5 to 12 years old and Old vines classified as 40 to 60 years old.

Old vines have been generally defined as those originally planted at least 50 years ago in California [8]; however, vines planted 35 years ago are considered the minimum age requirement for old vine designation in Australia [7]. Importantly, the relative age gap in this study between the youngest Old vines and the oldest Young vines are at least 28 years. A Control treatment representing the vine proportion in the block (2 to 1 ratio of Old to Young vines) was also included in order to account for differences in sugar accumulation and phenological progression. For harvest and winemaking, the Control treatment was based on tons, not vine proportion, in order to simulate a true harvest of the entire commercial block. For viticultural measurements, the Control treatment was measured based on vine proportion. However, pre-harvest viticultural measurements in 2019 were synthetically calculated using the existing Young and Old vine data because the Control treatment was added retroactively. The age of the vine was determined using visual identification; a root system as one year, a trunk and head as two to three years, an arm position as four years, a spur/shoot as five years, and every pre-existing spur position there after counted as another year. In 2020, composite dormant cane samples of Young vines and composite dormant cane samples of Old vines were found to be negative for Grapevine red blotch-associated virus (GRBaV), Grapevine leafroll-associated virus (GLRaV-1, GLRaV-2, GLRaV-3, GLRaV-4, GLRaV-5), Kober stem grooving virus (GVA), corky bark associated-virus (GVB), Grapevine Fanleaf Virus (GFIV), Pierce’s Disease (Xf), and Grapevine Pinot Gris virus (GPGV). Due to the prevalence of field blends in California old vine vineyards [52], classic ampelography was used to verify cv. Zinfandel vines. Grapes were harvested at a target Brix of 25 ± 0.5 normally indicated for standard (commercial) winemaking practices [53].

### 4.2. Climate Data

Weather data was obtained from California Irrigation Information Management System (CIMIS) station 163 (35°47′25.5″ N–120°64′81.4″ W), located 14.16 km from the experimental site. Precipitation and daily air temperatures were subsequently determined during the 2019 and 2020 growing season. Cumulative growing degree days (GDD) for seasonal (1 April to 31 October) and annual (1 January to 31 December) documentation were calculated using a baseline temperature of 10 °C and the daily average temperature [32].

### 4.3. Phenology and Senescence Tracking

Every two weeks, phenological tracking occurred on designated data collection vines in the block to measure key phenological events (i.e., budbreak, bloom, berry set, and harvest). The Modified Eichhorn-Lorenz (E-L) system was used to determine the numerical ranking of each vine [54]. In the 2019 growing season, only Young and Old vine treatments were measured (*n* = 12). In the 2020 growing season, a control treatment was added consisting of four randomly selected Young vines and eight randomly selected Old vines (*n* = 12).

In the 2020 growing season, the Dodson Walker Senescence scale was used to track leaf abscission and chlorosis on designated data collection vines every one to two weeks [30]. Numerical ranking was modified to track chlorosis and abscission separately due to the difference in progression (*n* = 12).

### 4.4. Leaf Water Potential and Gas Exchange Measurements

In the 2020 growing season, leaf gas exchange, including photosynthetic rate (A**_n_**) and stomatal conductance (g**_s_**), were measured with the LI-6400XT portable photosynthesis system (LICOR Biosciences, Lincoln, NE, USA) on mature and sun-exposed leaves. The LI-6400 parameters were set according to manufacturer recommendations. Internal photosynthetically active radiation (PARin) was set to equal the external photosynthetically active radiation (PARout). Reference CO_2_ was set at 400 mg/L, and the temperature and humidity set to reflect the ambient conditions. Immediately after leaf gas exchange measurements were completed, leaf water potential (Ψ_leaf_) readings were subsequently performed using the same leaf. This leaf was cut just above the thickness of the petiole with a razor blade, put in a plastic bag with the petiole upwards to limit water loss, and closely observed in a leaf pressure chamber to determine leaf water potential (PMS Instruments, Albany, OR, USA). Leaf gas exchange and subsequent leaf water potential measurements occurred diurnally on three replicate grapevines per treatment using one leaf per vine. Analysis occurred at véraison and four weeks post-véraison. Véraison measurements occurred on two consecutive days (114 and 155 days post-budbreak) due to significant difference in temperature. According to nearby CIMIS weather station 163, the maximum air temperature was 38.1 °C and 30.6 °C for each day, respectively. Pre-dawn Ψ_leaf_ and gas exchange measurements corresponded to those taken from 5:00 a.m. to 6:00 a.m., and mid-day Ψ_leaf_ and gas exchange measurements were taken from 11 a.m. to 3 p.m. Outliers were excluded from pre-dawn gas exchange measurements due to machine malfunction (*n* = 5 for Young vines at véraison day 2 and Control vines at véraison + 4; *n* = 6 for all treatments at véraison day 1).

In the 2019 growing season, Ψ_leaf_ measurements were taken at mid-day (11 a.m.–1 p.m.) from the newest, fully expanded, sun exposed leaves using a pressure chamber (PMS Instruments, Albany, OR, USA). A leaf was cut just above the thickness of the petiole with a razor blade and put in a plastic bag with the petiole upwards. Two leaves were measured per vine; if there was more than 0.1 MPa variation between the two leaves, the process was repeated on a third leaf (*n* = 6). Measurements were taken at berry formation and one week post-véraison. At the one-week post-véraison collection date (126 days post-budbreak), the Old vine treatment only had five vine replications. 

### 4.5. Statistical Analysis

All parametric statistical analyses were performed using JMP (SAS Institute, North Carolina, USA). Data was analyzed using a one-way ANOVA, two-way ANOVA, ANCOVA, and Tukey’s Honest Significant Difference test (HSD). Nonlinear regression analyses were performed with GraphPad Prism Version 9.0.0. Nonlinear least sum-of-squares estimation was used to find the regression that models the relationship between photosynthesis (A_n_) and stomatal conductance (g_s_) [%continuous variables, gs as covariate%]. An extra sum-of-squares F test was used to determine whether vine age treatments had distinct regressions. An additional ANCOVA was performed to support evidence of difference. Data transformation was performed as necessary to meet the assumptions of ANCOVA. Graphs were created on GraphPad Prism Version 9.0.0. 

## 5. Conclusions

This study evaluated the effect of vine age on phenology and gas exchange parameters in a single interplanted block with Young an Old vines over two consecutive growing seasons in cv. Zinfandel grown under dry farm conditions in the Central Coast of California. Vine age was found to have a significant impact on phenological timing in this study, with Young vines progressing slower during berry formation but quicker during berry ripening than Old vines in two consecutive seasons. Consequently, Young vines were harvested before Old vines due to significant differences in sugar accumulation (Table S2). Differences in harvest dates and growing season length could serve as a tool for wine growers in California; for example, younger vineyards may be less affected by late-season heatwaves. Related factors, such as vine balance, canopy architecture, and below-ground root architecture, should be investigated further to understand this trend. This trend is particularly important for interplanted vineyards with diverse age groups, as growing season length and sugar content has been shown to influence wine quality. Furthermore, significant differences in phenological timing and growing season length between age groups could warrant tailored vineyard management practices. Despite previous reports, in the present study there were no differences found in pre-dawn or mid-day Ψ_leaf_ measurements between Young and Old vines at differing phenological dates in the 2020 growing season (véraison and four weeks post-véraison). Additionally, no differences were found in mid-day Ψ_leaf_ measurements between Young and Old vines in the 2019 growing season. Young vines had higher mid-day stomatal conductance measurements, and tended to have higher mid-day photosynthetic rates, compared to Old vines. Logarithmic regression analysis of the relationship between mid-day photosynthetic rates and stomatal conductance found Young vines were statistically different from Old vines at véraison day 1, which was the warmest of the two dates. This suggests a difference in physiological behavior when exposed to heat stress; however, the underlying reasons for this response are unclear. This study is in accordance with others that highlight the need for special consideration of young vine establishment, particularly with an interplanted dry farmed vineyard in a warm climate. The results of this study indicate there is a difference in cv. Zinfandel vine phenology and gas exchange parameters between young (5 to 12 years old) and old (40 to 60 years old) vines grown in the Central Coast of California. This work is part of a larger comprehensive study to determine the effect of vine age on cv. Zinfandel grapes and wines. Importantly, this study shows vine age should be considered when evaluating the timing of phenological events. While the implications on subsequent wine quality in this study need to be further investigated, the perceived superior quality in “old vine” wines may be due to an extended berry ripening phase and longer growing season. 

## Figures and Tables

**Table 1:** Growing degree days (GDD); Winkler region classification; and precipitation for Atascadero, California (USA) weather station 163.

Growing Season.	Growing Degree Days (GDD) ^1^	Winkler Region	Annual Precipitation (mm) ^2^	Seasonal Precipitation (mm) ^3^
2019	1689.6	III	653.5	24.6
2020	1927.2	III	188.7	90.2

^1^ Calculated from 1 April–31 October in degree Celsius with a baseline of 10 °C. ^2^ Sum of precipitation from 1 January–31 December for 2019, and sum of precipitation from 1 January–21 December for 2020. ^3^ Sum of precipitation from 1 April–31 October.

**Table 2:** Monthly average air temperature, minimum air temperature, and maximum air temperature for Atascadero, California (USA) weather station 163 during the 2019 and 2020 growing season.

		2019			2020	
Month	Average Air Temperature (°C)	Minimum Air Temperature (°C)	Maximum Air Temperature (°C)	Average Air Temperature (°C)	Minimum Air Temperature (°C)	Maximum Air Temperature (°C)
April	14.0	6.3	22.5	13.7	6.1	21.5
May	13.5	6.9	21.0	16.8	7.4	26.3
June	18.4	9.9	28.0	18.7	9.6	28.3
July	20.6	10.7	31.5	19.5	9.8	30.1
August	21.1	11.8	32.1	22.1	12.5	33.1
September	19.0	9.4	30.1	20.5	10.0	33.2
October	14.0	2.9	27.1	16.9	6.9	29.9

**Table 3:** One-way analysis of variance (ANOVA) showing senescence (leaf chlorosis and abscission) tracking during the 2020 growing season (*n* = 12). Treatment means followed by standard error of the mean. Different letters within a column indicate differences between treatment groups based on Tukey HSD. Significant *p*-values (<0.05) are shown in the table.

Date	Treatment	Degree of Leaf Chlorosis	Degree of Leaf Abscission
	Young vines	1.083 ± 0.193 a	0.167 ± 0.167 a
10/18/20	Control	0.583 ± 0.149 a,b	0.000 ± 0.000 a
	Old vines	0.417 ± 0.149 b	0.000 ± 0.000 a
	*p*-value	0.0197	0.3788
	Young vines	1.333 ± 0.188 a	0.500 ± 0.261 a
10/30/20	Control	0.917 ± 0.083 b	0.500 ± 0.261 a
	Old vines	1.000 ± 0.000 a,b	0.167 ± 0.167 a
	*p*-value	0.0437	0.5151
	Young vines	6.000 ± 0.000 a	3.417 ± 0.229 a
11/13/20	Control	6.000 ± 0.000 a	3.750 ± 0.218 a
	Old vines	6.000 ± 0.000 a	3.917 ± 0.193 a
	*p*-value	1.0000	0.2563
	Young vines	6.000 ± 0.000 a	3.833 ± 0.345 a
11/22/20	Control	6.000 ± 0.000 a	4.417 ± 0.260 a
	Old vines	6.000 ± 0.000 a	4.417 ± 0.288 a
	*p*-value	1.0000	0.2955

**Table 4:** Two-way analysis of variance (ANOVA) with interaction showing mid-day leaf water potential (Ψ_leaf_) measurements by treatment and growth stage during the 2019 growing season (*n* = 6). Treatment means followed by standard error of the mean. Different letters within a column indicate significant differences between treatment groups based on Tukey HSD.

Growth Stage	Treatment	Mid-Day Ψ_leaf_ (MPa)
	Young	–1.230 ± 0.041 a
Berry Formation	Control	–1.219 ± 0.030 a
	Old	–1.225 ± 0.024 a
	*p*-value	0.9737
	Young	–1.283 ± 0.017 a
Véraison + 1 week	Control	–1.223 ± 0.035 a
	Old	–1.200 ± 0.032 a
	*p*-value	0.1409
Treatment (T)		0.3248
Growth Stage (G)		0.6754
T × G Interaction		0.4463

**Table 5:** Two-way analysis of variance (ANOVA) with interaction showing pre-dawn and mid-day leaf water potential (Ψ_leaf_) measurements by treatment and growth stage during the 2020 growing season (*n* = 6 and *n* = 15, respectively). Véraison + 4 corresponds to four weeks post-véraison. Treatment means followed by standard error of the mean. Different letters indicate significant differences between treatment groups based on Tukey HSD. Significant *p*-values (<0.05) are shown in the table.

Growth Stage	Treatment	Mid-day Ψ_leaf_ (MPa)	Pre-dawn Ψ_leaf_ (MPa)
	Young	–1.425 ± 0.021 a	–0.733 ± 0.092 a
Véraison Day 1	Control	–1.372 ± 0.024 a	–0.692 ± 0.132 a
	Old	–1.355 ± 0.024 a	–0.617 ± 0.119 a
	*p*-value	0.0915	0.7726
	Young	–1.280 ± 0.026 a	–0.454 ± 0.106 a
Véraison Day 2	Control	–1.257 ± 0.322 a	–0.504 ± 0.070 a
	Old	–1.230 ± 0.040 a	–0.421 ± 0.064 a
	*p*-value	0.5684	0.7737
	Young	–1.222 ± 0.036 a	–0.504 ± 0.068 a
Véraison + 4 weeks	Control	–1.257 ± 0.036 a	–0.558 ± 0.039 a
	Old	–1.222 ± 0.037 a	–0.550 ± 0.041 a
	*p*-value	0.7315	0.7270
Treatment (T)		0.2837	0.7331
Growth Stage (G)		<0.0001	0.0111
T × G Interaction		0.6754	0.9096

## Data Availability

All data pertaining to this project is being held in computers owned by California Polytechnic State University, San Luis Obispo, under control of the PI team.

## Supplementary Materials

The following are available online at https://www.mdpi.com/2223-7747/10/2/311/s1, Table S1: Vine Internode Length, Diameter, Yield, Cluster Number and Ravaz Index 2019 and 2020, Table S2: Harvest Berry Brix, pH and Titratable Acidity 2019 and 2020.
