# Supplementary material for: The Effect of Grapevine Age (Vitis vinifera L. cv. Zinfandel) on Phenology and Gas Exchange Parameters over Consecutive Growing Seasons
Source: Plants (Basel). 2021 Feb 5;10(2):311. doi: 10.3390/plants10020311 (PMC7915390; doi:10.3390/plants10020311)
Supplement: Supplementary file 1 [file plants-10-00311-s001.pdf]

Supplementary Materials

Table S1. Two-way analysis of variance (ANOVA) showing internode length, internode diameter, yield per vine, cluster number per vine, and Ravaz index from the 2019 and 2020 growing season (n = 12 for internode length and diameter; n = 7 and n = 15 for harvest parameters (yield per vine, cluster number per vine, and Ravaz index) in 2019 and 2020, respectively). Treatment means followed by standard error of the mean. Different letters indicate significant differences between treatment groups based on Tukey HSD. Significant p-values (<0.05) are shown in bold fonts.

Growing Season	Treatment	Internode Length (mm)	Internode Diameter (mm)	Yield per vine (kg)	Cluster Number per Vine (#)	Ravaz Index
	Young	62.86 ± 3.13 a	12.05 ± 0.46 a	3.49 ± 0.63 b	18.00 ± 2.23 b	4.160 ± 0.760 b
2019	Control	54.40 ± 3.13 ab	10.04 ± 0.46 b	7.46 ± 0.92 a	41.00 ± 3.92 a	6.389 ± 1.080 ab
	Old	48.49 ± 3.13 b	9.05 ± 0.46 b	7.03 ± 0.92 a	37.29 ± 3.24 a	7.982 ± 1.153 a
	p-value	**0.0100**	**0.0002**	**0.0062**	**0.0002**	**0.0485**
	Young	59.89 ± 1.87 a	11.67 ± 0.34 a	3.54 ± 0.50 b	17.67 ± 1.66 b	4.770 ± 0.584 b
2020	Control	47.65 ± 1.87 b	9.21 ± 0.34 b	5.18 ± 0.94 ab	33.53 ± 4.43 a	6.391 ± 0.907 b
	Old	42.96 ± 1.87 b	7.72 ± 0.34 c	7.38 ± 0.84 a	44.00 ± 3.76 a	10.206 ± 1.108 a
	p-value	**<0.0001**	**<0.0001**	**0.0047**	**<0.0001**	**0.0003**
**Treatment (T)**		**<0.0001**	**<0.0001**	**0.0004**	**<0.0001**	**0.0002**
**Growing Season (S)**		0.0146	0.0177	0.4020	0.9112	0.2746
**T x S Interaction**		0.745	0.5431	0.2894	0.2091	0.5532

Table S2. Two-way analysis of variance (ANOVA) showing sugar content (brix), pH, and titratable acidity (TA) at harvest from the 2019 and 2020 growing season. Treatment means followed by standard error of the mean. Different letters indicate significant differences between treatment groups based on Tukey HSD. Significant p-values (<0.05) are shown in bold fonts.

Growing Season	Treatment	Brix	pH	Titratable Acidity (TA) (g/L)
	Young	22.08 ± 0.42 b	3.41 ± 0.02 b	6.28 ± 0.32 b
2019	Control	24.05 ± 0.35 ab	3.45 ± 0.04 b	4.93 ± 0.24 c
	Old	25.17 ± 0.97 a	3.60 ± 0.03 a	7.21 ± 0.37 a
	p-value	**0.0129**	**0.0005**	**<0.0001**
	Young	24.50 ± 0.25 a	3.43 ± 0.04 a	6.38 ± 0.07 a
2020	Control	25.33 ± 0.67 a	3.35 ± 0.05 a	6.28 ± 0.16 a
	Old	24.08 ± 0.51 a	3.46 ± 0.03 a	5.99 ± 0.44 a
	p-value	0.2790	0.2298	0.6128
**Treatment (T)**		0.1053	**0.0024**	**0.0061**
**Growing Season (S)**		0.1446	**0.0149**	0.7443
**T x S Interaction**		0.0608	0.0637	**0.0008**
